# Utility of Th1-cell immune responses for distinguishing active tuberculosis from non-active tuberculosis: A case-control study

**DOI:** 10.1371/journal.pone.0177850

**Published:** 2017-05-22

**Authors:** Lifan Zhang, Xinhe Cheng, Sainan Bian, Yanhua Song, Qiang Li, Mengqiu Gao, Yueqiu Zhang, Xiaochun Shi, Xiaoqing Liu

**Affiliations:** 1 Division of Infectious Diseases, Peking Union Medical College Hospital, Chinese Academy of Medical Sciences & Peking Union Medical College, Beijing, China; 2 Clinical Epidemiology Unit, International Epidemiology Network, Peking Union Medical College Hospital, Chinese Academy of Medical Science, Beijing, China; 3 Centre for Tuberculosis Research, Chinese Academy of Medical Sciences and Peking Union Medical College, Beijing, China; 4 Tuberculosis Department, Beijing Chest Hospital, Capital Medical University, Beijing Tuberculosis and Thoracic Tumor Research Institute, Beijing, China; Chinese Academy of Medical Sciences and Peking Union Medical College, CHINA

## Abstract

Currently available Interferon-γ release assay (IGRA) cannot reliably differentiate active TB (ATB) from non-active TB (non-ATB). A study was performed to evaluate the value of Mycobacterium tuberculosis (MTB) specific Th1 cell immune responses which test IFN-γ and IL-2 simultaneous for differentiating ATB from non-ATB. Forty-nine newly diagnosed inpatients with ATB (26 pulmonary TB and 23 extrapulmonary TB) were enrolled as the ATB group. Forty-five volunteers with latent tuberculosis infection (LTBI) and twenty with evidence of previous TB were enrolled during the same period as the non-ATB group. Clinical examination and MTB specific Th1 cell immune responses were performed for all participants. After being stimulated with ESAT-6 and CFP-10, the median frequencies of single IL-2-, single IFN-γ-, and dual IFN-γ/IL-2-secreting T-cells were all higher in the ATB group than in the non-ATB group (20(8–45) vs. 7(3–13), P<0.001;131(44–308) vs. 10(6–27), P<0.001;25(9–74) vs. 7(3–23), P = 0.001, respectively). Evaluation of the diagnostic performance of detecting single IFN-γ-secreting T cells for pulmonary TB employed a cutoff value of 35 iSFCs/250,000 PBMC. The sensitivity, specificity, positive predictive value (PPV), negative predictive value (NPV), positive likelihood ratio (PLR), and negative likelihood ratio (NLR) were 92.3%, 80.0%, 64.9%, 96.3%, 4.62, and 0.10, respectively. For extrapulmonary TB, using a cutoff value of 23 iSFCs/ 250,000 PBMC, the sensitivity, specificity, PPV, NPV, PLR, and NLR were 91.3%, 76.9%, 58.3%, 96.2%, 3.96, and 0.11, respectively. When combining frequencies and proportion of single IFN-γ-secreting T cells, the test sensitivity was 100% in parallel tests and the specificity was 87.7% in serial tests for pulmonary TB. MTB specific Th1 cell immune responses (FluoroSpot) had value for the differentiation of ATB and non-ATB. Further confirmatory studies are indicated.

## Introduction

In 2014,9.6 million people were estimated to have developed tuberculosis (TB) worldwide, with an incidence of 133/100000, and 1.5 million people died of TB[1]. China is one of the 30 countries with the highest TB burdens, and also with a high burden of TB/HIV and multidrug resistance TB (MDR-TB). TB prevention remains a challenge to public health. According to WHO’s estimation, there were 930000 new TB cases in China in 2014, accounting for 10% of worldwide cases, following only India and Indonesia[1]. The fifth national tuberculosis epidemiological survey of China in 2010 showed that the prevalence of active pulmonary TB was459/100000 among people over 15 years old [2].

Diagnosis of tuberculosis remains unsatisfactory. The traditional diagnostic methods have a number of notable shortcomings, such as the low detection rate of acid-fast smear, a long turnaround time of culture of Mycobacterium tuberculosis (MTB), and the invasiveness of histopathological examination. As tuberculin purified protein derivative (PPD) includes the common antigenic components of non-tuberculous mycobacterium and Bacillus Calmette-Guérin (BCG), the “false positive rate” is high in areas where vaccination of BCG is widely implemented[3]; while the sensitivity is low among immunocompromised individuals who are more susceptible to tuberculosis infection than the general population[4].

The appearance of the interferon-γ release assay (IGRA) assay is considered to have created a new era in the diagnosis of tuberculosis infection[5]. In recent years, IGRA has been widely recognized and used in clinical practice[3,6–8]. IGRA is based on MTB-specific T cell responses, and detects the secretion of the cytokine IFN-γ. However, studies have shown that it is difficult to differentiate active TB (ATB) from latent TB infection (LTBI) using IGRA[9]. Previous studies of IGRA for the diagnosis of TB conducted by our team suggest that specificity of T-SPOT. TB was only on the order of70% for diagnosis of ATB[10,11]. The multicenter epidemiological investigation of LTBI in China in 2013 reported that the positive rate of IGRA (QFT-GIT) was 18.9% in rural China, which indicated that nearly 20% of the general population in China had LTBI[12]. In high TB prevalence areas such as China, high LTBI would inevitably reduce the specificity of IGRA for the diagnosis of ATB.

Similar to IFN-γ, IL-2 is another cytokine secreted by Th1 lymphocytes, which are involved in cellular immunity as caused by MTB. It has been shown that, IL-2 and IFN-γ secreted by antigen-specific T cells changed according to different TB infection status; the number of IL-2 secreting T cells in LTBI was higher than that in ATB patients, indicating that IL-2 might serve as the appropriate biomarker to distinguish different clinical stages of human tuberculosis infection[7,13].

The development of Fluorescence-Immunospot (FluoroSpot) assay technology was based on the traditional ELISPOT method. Compared to traditional detection, FluoroSpot technology can simultaneously detect two or more cytokines on the level of single cells. This technique may enhance the application of ELISPOT technology. Preliminary studies have found that detecting IFN-γ and IL-2 secretion by antigen specific T cells with the FluoroSpot method might distinguish different clinical stages of tuberculosis infection[14],but relevant data is limited.

This study evaluated the clinical application value of the FluoroSpot assay which simultaneously detects IFN-γ and IL-2 secreted by Th1 lymphocytes for the differential diagnosis of ATB and non-ATB.

## Methods

### Patient selection and study process

For the ATB group, patients (age ≥16 years old) with smear-positive pulmonary TB (PTB) were recruited in Beijing Chest Hospital, and patients (age ≥16 years old) with extrapulmonary TB (EPTB) were enrolled in Peking Union Medical College Hospital (PUMCH) from December 2012 to December 2015. For the non-ATB group, healthcare workers (LTBI without other diseases) were enrolled voluntarily from both PUMCH and Beijing Chest Hospital, and LTBI patients with other diseases were included in PUMCH. Additionally, patients with evidence of previous TB were included in the PUMCH during the study period. Clinicians who made the final diagnosis for each patient were blinded to the results of FluoroSpot.

The inclusion criteria were as the following:

Pulmonary TB (PTB): 1) With clinical manifestations such as fever, cough, chest pain, and manifestations of chest radiogram; AND 2) Positive results of sputum smear acid-fast stain or culture of MTB of sputum, AND 3) Untreated or received anti-TB treatment within 4 weeks.

Extra-pulmonary TB (EPTB): 1) With clinical manifestations such as fever, night sweats, weight loss, and corresponding symptoms of the related organ; AND 2) Positive results of smear acid-fast stain or culture of MTB, OR typical histological manifestation of TB (such as caseous necrosis and epithelioid granuloma, etc); AND 3) Untreated or received anti-TB treatment within 4 weeks.

Latent TB infection (LTBI): 1) With no clinical manifestations of active TB including fever, cough, chest pain; AND 2) Had no medical history of TB, no manifestations of previous TB in chest radiogram; AND 3) Positive result of T-SPOT.TB.

Previous TB: 1) Previous history of TB OR radiological manifestation of previous TB; AND 2) Clinical manifestations can be due to other diseases; AND 3) Positive T-SPOT.TB.

The protocol was approved by the Institutional Review Board of Peking Union Medical College Hospital. This study didn’t include minors, and each participant enrolled in this study provided written informed consent. Patient information was de-identified prior to analysis.

### FluoroSpot method

**Fig 1:**
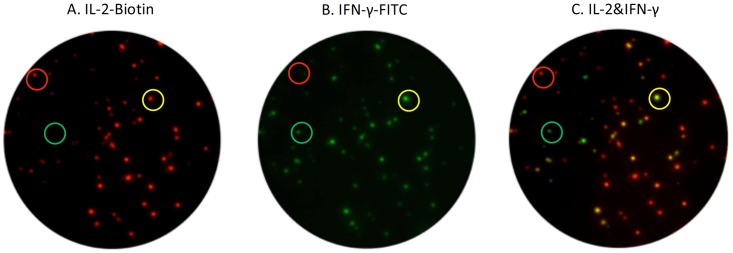
The dual-color Fluorospot assay detecting single IFN-γ-, single IL-2-, and dual IFN-γ/IL-2-secreting T cells.

**Fig 2:**
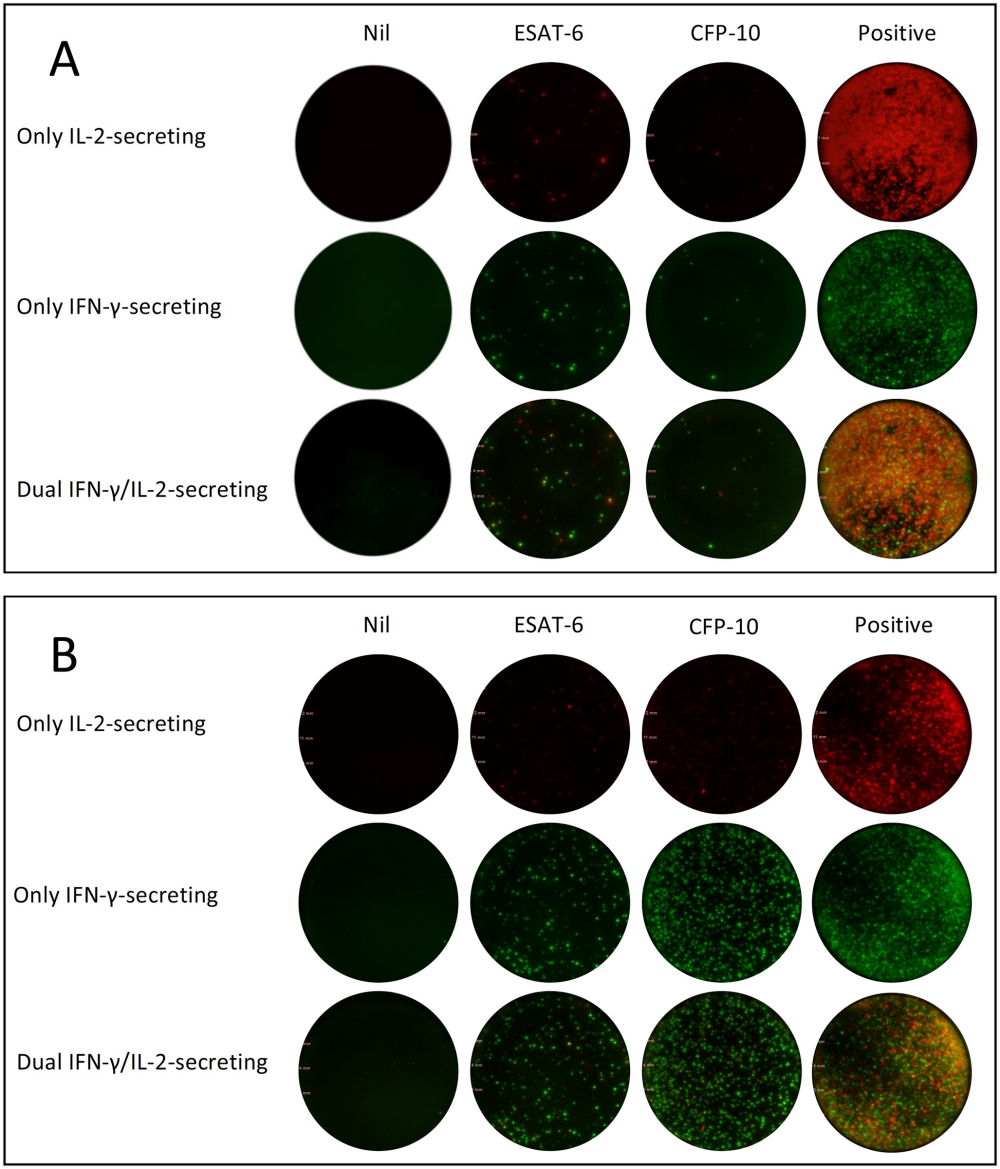
Examples of the results of the dual-color Fluorospot assay. (A) The results of the Fluorospot assay of a patient with ATB. (B) The results of the Fluorospot assay of a patient with LTBI.

Eight milliliter of peripheral blood was collected from each participant. FluoroSpot (AID, Straßberg, Germany) detection was performed within six hours from sample collection by laboratory staff blinded to patients’ clinical data. Peripheral blood mononuclear cells (PBMC) were isolated by Ficoll-Hypaque gradient centrifugation. 96-well plates were pre-coated with monoclonal antibodies against interferon γ (IFN-γ) and interleukin 2 (IL-2). Duplicate wells were seeded with 2.5×10^5^ PBMCs and anti-CD28 (0.5 μg/mL, AID, Straßberg, Germany) and contained: AIM-V (GIBCO^™^ AIM V Medium liquid, Invitrogen, US.) as negative control, 5 μg/ml PHA as positive control, and peptides of ESAT-6 or CFP-10 (final concentration of each peptide was 10μg/ml) as specific antigens, respectively. Plates were incubated 18–20 hours at 37°C in 5% carbon dioxide, and then washed with washing buffer. After washing, plates were incubated with IFN-γ-FITC and IL-2-biotin tagged with fluorescein, and the plates were incubated with fluorophore-labelled secondary antibodies and fluorescent enhancer. Specific T cell secreting single IFN-γ-, single IL-2-, and dual IFN-γ/IL-2 were counted by an automated fluorescence plate reader (AID-iSpot, Straßberg, Germany).(Figs 1 and 2).

### Statistical analysis

Sensitivity, specificity, predictive value (PV) and likelihood ratio (LR) were calculated to evaluate diagnostic accuracy for the FluoroSpot assay. Receiver operating characteristics (ROC) curves were developed to establish optimal cutoffs of the FluoroSpot method to differentiate ATB from non-ATB. Variables of normal distribution are expressed as mean±SD, variables of non-normal distribution are expressed as median and IQR. Enumeration data are expressed as percentage and 95% confidence interval (CI). Continuous variables of normal distribution were assessed using Students’ t-tests, and those of non-normal distribution were assessed by Mann-Whitney U test. The Pearson’s Chi-square test was used to compare proportions. Significance was inferred for P <0.05 (two-sided). Statistical analyses were performed using SPSS16.0 (SPSS Inc, USA).

## Results

Forty-nine patients with ATB were included in the study, with 26 patients of smear-positive PTB, and 23 of EPTB.45 subjects with LTBI (24 without other diseases and 21 with other diseases) and 20 patients with evidences of previous TB were included in the study. The details are shown in Table 1.

**Table 1:** Demographic and characteristics of the study subjects.

	PTB (n = 26)	EPTB^a^ (n = 23)	Previous-TB^b^ (n = 20)	LTBI (without other diseases) (n = 24)	LTBI (with other diseases)^c^ (n = 21)	Total (n = 114)
Age(years),(Mean± SD)	37±13	44±18	39±20	34±6	41±18	39±15
Gender, Male (n, %)	23(88.5)	8(34.8)	8(40.0)	4(16.7)	10(47.6)	53(46.5)

PTB: Pulmonary tuberculosis; EPTB: Extrapulmonary tuberculosis; LTBI: Latent tuberculosis infection; ATB: Active tuberculosis

^a^. The 23 cases of extrapulmonary TB include 8 cases of tuberculous lymphadenitis, 5 cases of intestinal tuberculosis, 2 cases of genital tuberculosis, 1 case of hepatic tuberculosis, 1 case of cutaneous tuberculosis, 1 case of mammary tuberculosis, 1 case of renal tuberculosis, 1 case of esophageal tuberculosis, 1 case of tuberculous endocarditis, 1 case of tuberculosis of the eyelid, and 1 case of tuberculous otitis media.

^b^. The 20 cases with evidence of previous TB include systemic vasculitis (n = 2), systemic lupus erythematosus (n = 1), mixed connective tissue disease (n = 1), polymyalgia rheumatica (n = 1), Takayasu arteritis (n = 1), brucellosis (n = 1), infection of the upper respiratory tract (n = 1), pneumonia (n = 1), cured TB (n = 1), endometrial stromal sarcoma (n = 1), lung cancer (n = 1), esophageal carcinoma (n = 1), coronary heart disease (n = 1), oral ulcer (n = 1), histiocytic necrotic lymphadenitis (n = 1), dermatitis (n = 1), chronic uveitis (n = 1), cerebellar ataxia (n = 1), primary infertility (n = 1).

^c^.The twenty-one cases of LTBI with other diseases were diagnosed with lung cancer (n = 3), Takayasu arteritis (n = 2), ulcerative colitis (n = 2), pneumonia (n = 1), urinary tract infection (n = 1), asthma (n = 1), systemic lupus erythematosus (n = 1), renal artery stenosis (n = 1), carcinoma of renal pelvis (n = 1), nephrotic syndrome (n = 1), Behcet disease (n = 1), Type 2 diabetes mellitus (n = 1), adrenal cortical hyperplasia (n = 1), chronic renal failure (n = 1), obstructive jaundice (n = 1), endometriotic cyst (n = 1),and lower extremity atherosclerotic occlusive disease (n = 1).

### Comparison of frequencies of MTB specific IFN-γ and IL-2 secreting T cells

After being stimulated with the antigens ESAT-6, CFP-10, or both the two antigens, frequencies of singleIL-2-, single IFN-γ-, and dual IFN-γ/IL-2-secreting T-cells were all higher in the ATB group than in the non-ATB groups (P<0.05).

PTB patients also had significantly higher frequencies of single IFN-γ-secreting T-cells in comparison to EPTB patients (P<0.05).

There were no significant differences between the LTBI and the previous TB groups (Table 2).

**Table 2:** The frequencies of ESAT-6- and CFP-10-specific single IFN-γ-, single IL-2-, and dual IFN-γ/IL-2-secreting T cells in patients with ATB and non-ATB (iSFCs/250,000 PBMC).

Antigens	Cytokines (Median, IQR)	PTB	EPTB	P value	LTBI(without other diseases)	LTBI(with other diseases)	Previous-TB	P value	ATB	Non-ATB	P value
ESAT-6	Single IL-2	16(8–27)	17(8–33)	0.527	4(3–10)	4(2–7)	3(2–6)	0.600	17(8–30)	4(2–7)	<0.001
	Single IFN-γ	101(35–237)	27(21–54)	0.015	7(4–24)	11(1–18)	6(2–12)	0.510	52(23–167)	7()3-18)	<0.001
	Dual	12(5–25)	21(5–35)	0.171	8(2–19)	2(1–15)	8(2–18)	0.190	15(5–29)	6(2–16)	0.006
CFP-10	Single IL-2	8(3–26)	9(5–31)	0.480	4(2–7)	3(2–16)	5(2–12)	0.813	8(3–27)	4(2–8)	0.034
	Single IFN-γ	105(29–183)	36(5–83)	0.009	5(2–25)	5(2–33)	5(2–9)	0.966	62(8–132)	5(2–18)	<0.001
	Dual	7(3–25)	29(3–59)	0.187	8(1–19)	3(1–22)	3(2–5)	0.756	8(3–40)	4(2–13)	0.010
ESAT-6&CFP-10	Single IL-2	19(7–43)	22(8–69)	0.637	7(5–14)	7(3–16)	5(3–13)	0.813	20(8–45)	7(3–13)	<0.001
	Single IFN-γ	248(81–371)	66(30–155)	0.004	10(7–50)	13(2–20)	8(6–16)	0.704	131(44–308)	10(6–27)	<0.001
	Dual	20(8–37)	45(14–117)	0.115	8(3–34)	2(1–15)	11(6–19)	0.103	25(9–74)	7(3–23)	0.001

PTB: Pulmonary tuberculosis; EPTB: Extrapulmonary tuberculosis; LTBI: Latent tuberculosis infection; ATB: Active tuberculosis

### Comparison of proportion of MTB specific IFN-γ and IL-2 secreting T cells

After stimulated with antigen ESAT-6, the median proportion of single IFN-γ-secreting T-cells in patients with ATB and non-ATB was 0.69(IQR 0.42–0.86) and 0.50(IQR 0.28–0.78), respectively (P = 0.039). After stimulated with antigen CFP-10, the median proportion of single IFN-γ-secreting T-cells was 0.75(IQR 0.53–0.89) in the ATB group, which was significantly higher than in the non-ATB group (0.50(IQR 0.39–0.71), P = 0.002). Furthermore, there were no significant differences between the two groups in proportion of single IL-2-and dual IFN-γ/IL-2-secreting T cells (Table 3).

**Table 3:** The proportion of ESAT-6 and CFP-10 specific single IFN-γ, single IL-2, and dual IFN-γ/IL-2 secreting T cells in patients with ATB and non-ATB (iSFCs/250,000 PBMC).

Antigens	Cytokines (Median, IQR)	PTB	EPTB	P value	LTBI(without other diseases)	LTBI(with other diseases)	Previous-TB	P value	ATB	Non-ATB	P value
ESAT-6	Single IL-2%	0.07(0.04–0.12)	0.20(0.05–0.29)	0.052	0.13(0–0.41)	0.13(0–0.30)	0.14(0–0.40)	0.862	0.10(0.04–0.20)	0.13(0–0.38)	0.473
	Single IFN-γ%	0.78(0.66–0.89)	0.42(0.34–0.71)	0.002	0.57(0.29–0.83)	0.52(0.35–0.91)	0.36(0.22–0.67)	0.127	0.69(0.42–0.86)	0.50(0.28–0.78)	0.039
	Dual%	0.08(0.05–0.20)	0.31(0.06–0.40)	0.045	0.17(0–0.32)	0.11(0–0.48)	0.35(0.14–0.53)	0.118	0.10(0.05–0.35)	0.22(0–0.45)	0.539
CFP-10	Single IL-2%	0.05(0.01–0.14)	0.15(0.03–0.27)	0.068	0.14(0–0.37)	0.20(0–0.60)	0.20(0–0.43)	0.653	0.08(0.01–0.18)	0.20(0–0.41)	0.082
	Single IFN-γ%	0.80(0.68–0.91)	0.57(0.47–0.78)	0.009	0.52(0.45–0.82)	0.40(0.16–0.77)	0.44(0.31–0.60)	0.189	0.75(0.53–0.89)	0.50(0.39–0.71)	0.002
	Dual%	0.05(0.02–0.25)	0.24(0.08–0.37)	0.051	0.14(0–0.37)	0.02(0–0.49)	0.19(0.12–0.44)	0.233	0.15(0.03–0.32)	0.15(0–0.41)	0.769
ESAT-6&CFP-10	Single IL-2%	0.07(0.04–0.11)	0.13(0.07–0.26)	0.014	0.17(0.01–0.35)	0.21(0–0.44)	0.25(0.09–0.34)	0.895	0.09(0.05–0.18)	0.21(0.04–0.34)	0.033
	Single IFN-γ%	0.80(0.69–0.90)	0.46(0.44–0.77)	0.001	0.58(0.41–0.82)	0.58(0.32–0.80)	0.37(0.31–0.63)	0.138	0.74(0.46–0.86)	0.48(0.34–0.75)	0.007
	Dual%	0.06(0.04–0.21)	0.30(0.10–0.38)	0.001	0.16(0–0.33)	0.11(0–0.28)	0.30(0.16–0.47)	0.107	0.16(0.05–0.30)	0.19(0.04–0.36)	0.616

PTB: Pulmonary tuberculosis; EPTB: Extrapulmonary tuberculosis; LTBI: Latent tuberculosis infection; ATB: Active tuberculosis

After stimulated with both ESAT-6 and CFP-10, the median proportion of single IFN-γ-secreting T-cells in patients with ATB was 0.74 (IQR 0.46–0.86), which was significantly higher than that in non-ATB patients (0.48 (IQR 0.34–0.75), P = 0.007). The median proportion of singleIL-2-secreting T-cells in the ATB group was significantly lower than that in the non-ATB group (0.09(IQR 0.05–0.18) vs. 0.21(IQR 0.04–0.34), P = 0.033).

PTB patients had significant higher proportion of single IFN-γ-secreting T-cells in comparison to EPTB patients (P<0.05). There were no significant differences between LTBI and previous TB group.

### Differentiating ATB from non-ATB

**Fig 3:**
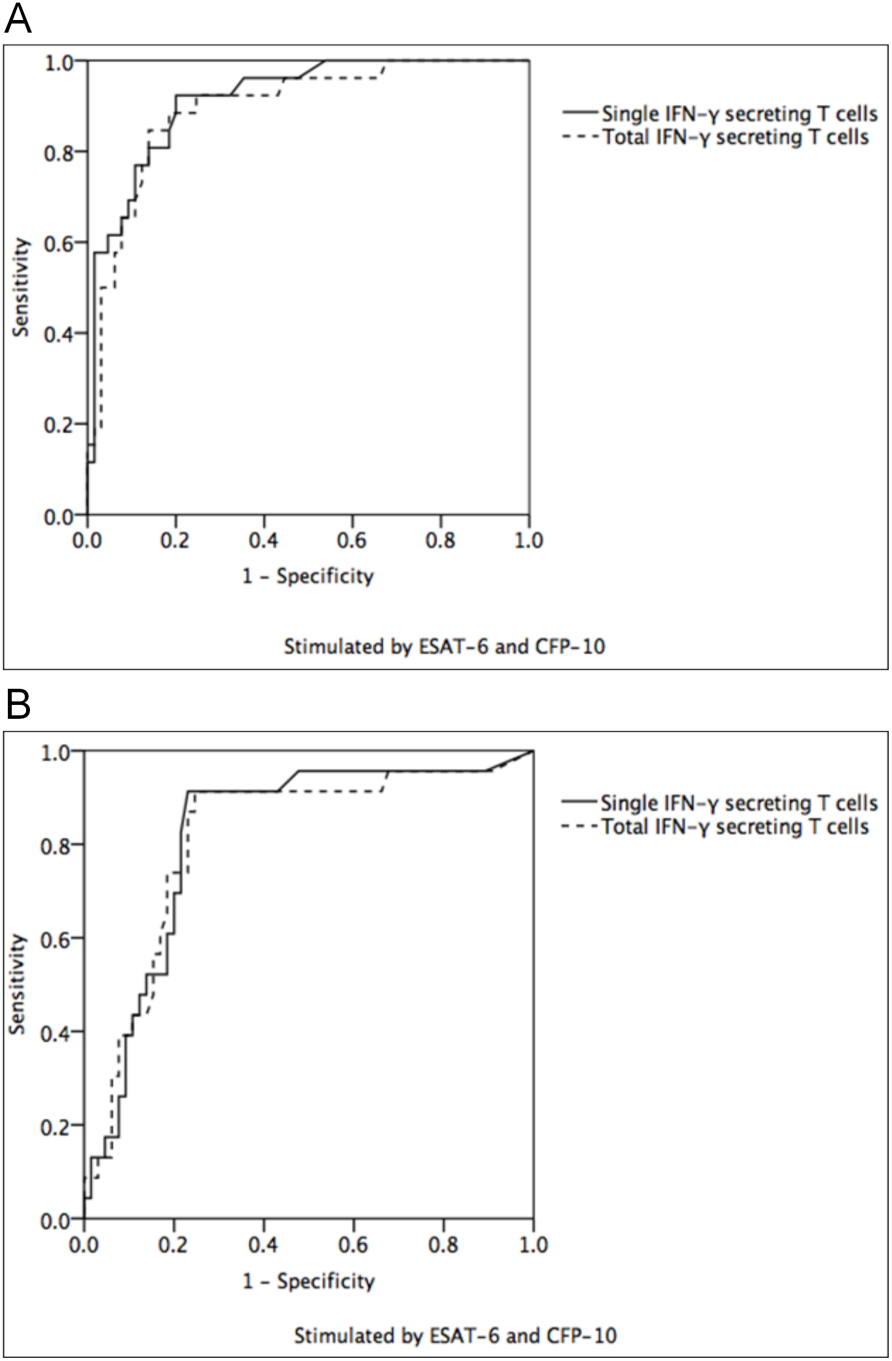
ROC curve for differentiating ATB from non-ATB. **(A)** For PTB, the area under the ROC curve was the largest when detecting single IFN-γ-secreting T cells, which was 0.916 (95% CI: 0.857–0.976). With the cutoff value of 35 iSFCs/ 250,000 PBMC, sensitivity was 92.3% and specificity was 80.0%. **(B)** For EPTB, the area under the ROC curve was the largest when detecting single IFN-γ-secreting T cells, which was 0.820 (95% CI: 0.720–0.920). With the cutoff value of 23 iSFCs/ 250,000 PBMC, sensitivity was 91.3% and specificity was 76.9%.

By ROC curve analysis, after stimulated by ESAT-6and CFP-10, the optimal cutoff value, sensitivity, specificity and area under ROC curve (AUROC) to differentiate ATB from non-ATB are shown in Fig 3.

For PTB, stimulated by both ESAT-6 and CFP-10, the AUROC was 0.916 (95%CI: 0.857–0.976) when detecting single IFN-γ-secreting T cells, which was larger than that when detecting total IFN-γ-secreting T cells (0.896, 95%CI:0.825–0.967). With the cutoff value of 35 iSFCs/ 250,000 PBMC, sensitivity was 92.3% and specificity was 80.0% (Fig 3A).

With a combination of frequencies and proportion of single IFN-γ-secreting T cells, parallel test raised sensitivity from 92.3% to 100%, and serial test raised specificity from 80.0% to 87.7%.

For EPTB, stimulated by both ESAT-6 and CFP-10, the AUROC was 0.820 (95% CI: 0.720–0.920) when detecting single IFN-γ-secreting T cells, which was similar to that when detecting total IFN-γ-secreting T cells (0.814,95%CI:0.709–0.920). With the cutoff value of 23 iSFCs/ 250,000 PBMC, sensitivity was 91.3% and specificity was 76.9% (Fig 3B).

With a combination of frequencies and proportion of single IFN-γ-secreting T cells, parallel test raised sensitivity from 91.3% to 95.7%, and serial test raised specificity from 76.9% to 81.3%.

The diagnostic value of FluoroSpot method for diagnosis of PTB and EPTB is shown in Tables 4 and 5, respectively.

**Table 4:** Diagnostic value of FluoroSpot method for diagnosis of pulmonary TB when stimulated by both ESAT-6 and CFP-10.

	Sensitivity (95%CI)	Specificity (95%CI)	PLR (95%CI)	NLR (95%CI)	PPV (95%CI)	NPV (95%CI)
Frequencies of single IFN-γ-secreting T cells	92.3(74.9–99.1)	80.0(68.2–88.9)	4.62(2.80–7.60)	0.10(0.03–0.37)	64.9(47.5–79.8)	96.3(87.3–99.6)
Proportion of single IFN-γ-secreting T cells	96.2(80.4–99.9)	61.7(48.2–73.9)	2.51(1.80–3.49)	0.06(0.01–0.43)	52.1(37.2–66.7)	97.4(86.2–99.9)
Parallel test	100.0(86.8–100.0)	56.9(44.0–69.2)	2.32(1.76–3.07)	/	48.2(34.3–62.2)	100.0(90.5–100.0)
Serial test	88.5(69.9–97.6)	87.7(77.2–94.5)	7.19(3.70–13.96)	0.13(0.05–0.38)	74.2(55.4–88.1)	95.0(86.1–99.00)

**Table 5:** Diagnostic value of FluoroSpot method for diagnosis of extrapulmonary TB when stimulated by both ESAT-6 and CFP-10.

	Sensitivity %(95CI)	Specificity %(95CI)	PLR (95CI)	NLR (95CI)	PPV %(95CI)	NPV %(95CI)
Frequencies of single IFN-γ-secreting T cells	91.3(72.0–98.9)	76.9(64.8–86.5)	3.96(2.49–6.28)	0.11(0.03–0.43)	58.3(40.8–74.5)	96.2(86.8–99.5)
Proportion of single IFN-γ-secreting T cells	77.3(54.6–92.2)	46.7(33.7–60.0)	1.45(1.04–2.01)	0.49(0.20–1.10)	34.7(21.7–49.6)	84.9(68.1–94.9)
Parallel test	95.7(78.1–99.9)	46.2(33.7–59.0)	1.78(1.40–2.26)	0.09(0.01–0.65)	38.6(26.0–52.4)	96.8(83.3–99.9)
Serial test	69.6(47.1–86.8)	81.3(69.5–89.9)	3.71(2.08–6.61)	0.37(0.20–0.70)	57.1(37.2–75.5)	88.1(77.1–95.1)

## Discussion

This study was performed to evaluate the diagnostic value of IFN-γ and IL-2 by FluoroSpot method in the differentiation of ATB from non-ATB in China. We evaluated the frequencies and proportion of single IFN-γ-, singleIL-2-, and dual IFN-γ/IL-2-secreting T cells in patients with ATB (pulmonary and extrapulmonary), LTBI and previous TB and analyzed the irrespective differential diagnostic values.

IFN-γ release assays (IGRA) which diagnoses tuberculosis infection by detecting IFN-γ are based on MTB-specific T cell responses, and have been listed in practice guidelines in many countries. Studies show that frequencies of IFN-γ secreting specific T cells in ATB patients were significantly higher than those in LTBI patients, however, the considerable overlap in the two groups made it difficult to distinguish ATB from LTBI[15,16]. Bittelet al[17] reported that in Switzerland ATB patients with no treatment had predominantly single IFN-γ-secreting T cells; ATB patients with anti-TB treatment had equal numbers of single IFN-γ-and singleIL-2-secreting T cells and asymptomatic medical workers had predominantly single IL-2-secreting T cells. The detection of IFN-γ and IL-2cytokine profiles by FluoroSpot is expected to distinguish ATB from LTBI.

In this study, ATB patients were either untreated or treated for less than four weeks. In ATB patients, frequencies of single IFN-γ-, singleIL-2- and dual IFN-γ/IL-2-secreting T cells were significantly higher than in non-ATB patients. The study by Chesov, et al[18] from Germany showed different results from our study in that only after stimulation by ESAT-6 were frequencies of single IFN-γ secreting T cells higher in ATB patients than in LTBI patients, and no differences were observed of other single IL-2 and dual IFN-γ and IL-2 secreting T cells between the two groups. Similar to this study, Chesov’s study also showed no significant difference between patients with previous TB and patients with LTBI. Essone PN et al[19] in South Africa also found that frequencies of single IFN-γ secreting T cells were higher in the TB group than in the healthy control group (P = 0.062).

After being stimulated with ESAT-6 and CFP-10, frequencies of single IL-2-, single IFN-γ- and dual IFN-γ/IL-2-secreting T-cells were all higher in the ATB group than in the non-ATB group, which may prove helpful in the differential diagnosis of ATB and non-ATB.

Interestingly, we discovered that both frequencies and proportion of single IFN-γ-secreting T-cells in patients with PTB were higher than those with EPTB. There might be two explanations. First, all patients with PTB had positive sputum smear acid-fast stain or culture of MTB, while in patients with EPTB, only 3 patients were pathogen positive. There active intensity might be stronger in pathogen positive patients than in pathogen negative patients. Second, more patients in PTB group were untreated than EPTB group, and anti-TB therapy might decrease the intensity of the IGRA reaction. This needs to be confirmed in further studies.

The non-ATB participants in this study were all T-SPOT.TB positive. Based on this, the ROC curve analysis showed that after stimulated by both ESAT-6 and CFP-10, the detection of frequencies of single IFN-γ-secreting T cells had the largest area under ROC curve and had the greatest differential diagnostic value, especially for active PTB, with the sensitivity of 92.3% and specificity of80.0%.

Furthermore, it was meaningful that with a combination of frequencies and proportion of single IFN-γ-secreting T cells, the diagnostic value could be increased. For PTB, sensitivity and specificity went up 7.7% with parallel and serial test, respectively. For EPTB, sensitivity and specificity went up 4.4% with parallel and serial test, respectively. Therefore, among the population with positive T-SPOT.TB, specificity for diagnosis of ATB was over 80% (87.7% for PTB, and81.3% for EPTB), which is of significance for high TB epidemic countries.

There are two main limitations of this study: 1) the study design was case-control, and thus might overestimate the differential diagnostic value of FluoroSpot in ATB and non-ATB; 2) we did not simultaneously detect T-SPOT.TB, and were unable to evaluate the consistency of T-SPOT.TB and FluoroSpot, but previous studies had confirmed good agreement[14].

In summary, based on the positive IGRA results, simultaneous detection of IFN-γ and IL-2 by FluoroSpot has value in the differential diagnosis of ATB and non-ATB. A combination of frequencies and proportion of single IFN-γ-secreting T cells is most effective for differential diagnosis
